# Time dynamics of elevated glucose and beta-hydroxybutyrate on beta cell mitochondrial metabolism

**DOI:** 10.1080/19382014.2025.2503515

**Published:** 2025-05-19

**Authors:** Ik Hals, Z. Ma, M. Kylling, A. Bjørkvik, A. Zhao, S-B. Catrina, X. Zhang, A. Björklund, V. Grill

**Affiliations:** a Department of Clinical and Molecular Medicine, Faculty of Medicine and Health Sciences, Norwegian University of Science and Technology (NTNU), Trondheim, Norway; b Department of Research, Nord-Trøndelag Hospital Trust, Levanger, Norway; c Department of Molecular Medicine and Surgery, Karolinska Institutet, Stockholm, Sweden; d Academic Specialist Center, Center for Diabetes, Stockholm, Sweden

**Keywords:** Beta-hydroxybutyrate, elevated glucose, INS-1 832/13 cells, mitochondrial metabolism, oxygen consumption rate, rat pancreatic islets

## Abstract

Chronic hyperglycemia impairs mitochondrial function of beta cells. Changes in mitochondrial function preceding a negative glucose effect have not been fully characterized, nor interactions with ketones. To compare effects on beta cell mitochondrial function by short and longer exposures to elevated glucose and interactions with ketones oxygen consumption rate (OCR) was measured in intact clonal beta cells by an OROBOROS and in rat islets by a Seahorse instrument. Proteins (subunits) of mitochondrial complexes (C) were measured by immunoblotting. ATP and ROS were measured in islets. In INS-1 832/13 cells, overnight exposure to 27 vs. 11 mm glucose increased OCR and uncoupled mitochondrial respiration. These effects vanished when prolonging the exposure time of elevated glucose. C1 was decreased after two days of culture with high glucose. Interactions with racemic 5 and 20 mm beta-hydroxybutyrate (BHB) were not detected. In islets, culture overnight at 27 vs.11 mm glucose enhanced basal OCR. No decrease in glucose-induced OCR was seen after prolonging 27 mm glucose for two days. Interactions with 5 mm BHB were not detected. Prolonged exposure to 27 mm glucose enhanced basal ECAR (extracellular acidification rate) and an ECAR response to acute elevation of glucose. C1 and 3 and 4 were decreased after two days of 27 vs. 11 mm glucose. ATP levels were decreased at this time-point and extracellular ROS increased. High glucose time-dependently affects mitochondrial function in clonal beta cells and islets. C1 was uniformly decreased. Interactions with BHB were not detected.

## Graphical abstract

**Figure:**
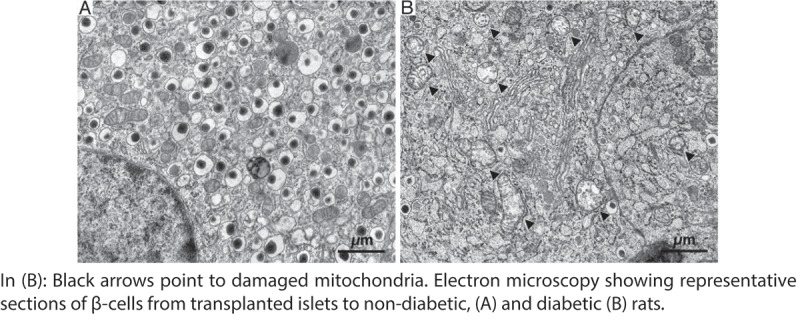

## Introduction

Type 2 diabetes tends to progress with increasing duration of the disease. Progression is manifested as a worsening of metabolic control which in turn is usually secondary to deterioration of insulin-producing beta cells. Chronic hyperglycemia has been implicated as a major factor behind the successive demise of beta cells;^1^ many mechanisms behind the impact of hyperglycemia on these cells have been proposed, but consensus on causality has not been reached.

Normal mitochondrial metabolism is essential for providing the energy needed for insulin secretion and signs of mitochondrial dysfunction are associated with beta cell demise.^2^ A previous study from our group demonstrated both morphological and functional abnormalities in beta cell mitochondria from streptozotocin-diabetic rats.^3^ Further, beta cell mitochondrial metabolism was shown to be severely perturbed in a mice model of diabetes after 2–3 week of culture at high glucose.^4^

Mitochondria are under physiological conditions the major organelle that processes glucose in cells, including beta cells, and mitochondria have evolved to handle day-to-day fluctuations of nutrients. We reasoned that the challenge of increased levels of glucose could initially lead to suitable adaptations of mitochondrial function and to frank dysfunction only after continued exposure to elevated glucose. However, the timeline for a switch from adaption to dysfunction and the nature thereof has not been investigated in detail.

Here we wished to elucidate the early time dynamics of beta cell mitochondrial function in response to variable exposure times to elevated glucose. To this end we subjected clonal beta cells and rat pancreatic islets to culture with elevated glucose either for overnight or 2–3 days and measured functional responses by respirometry, by impact on mitochondrial complexes and by viability and ROS parameters. Ketones are markedly elevated during a stage of untreated diabetes with hyperglycemia,^5^ however a paucity of studies have investigated mitochondrial interactions of ketones with elevated glucose. Hence, our investigations included measurements of a possible impact on glucose effects by beta-hydroxybutyrate at concentrations to be encountered during ketoacidosis.^5^

## Methods

### For basic experimental design see supplemental Figure S1.

#### INS-1 832/13 cells

INS-1 832/13 cells^6^ were used for experiments within passages 45–59. The cells were grown in 75 cm^2^ flasks in 10–11 mL medium. The medium used for culture (prior to experimental protocols) consisted of RPMI 1640 R8758 (Sigma-Aldrich) with 11 mm glucose concentration, supplemented with fetal calf serum (10%), HEPES (10 mm), sodium pyruvate (1 mm), penicillin (100 IU/mL), streptomycin (100 µg/mL), β-Mercapto-ethanol (50 µM) and L-glutamine (4 mm). The cells were incubated in a humidified incubator with 95% air and 5% CO_2_ at 37°C. The medium was changed every three to four days and the cells were sub-cultured once a week. Trypsin (0.01%) in ethylenediaminetetraacetic acid (EDTA) (0,02%) was used to detach the cells from the bottom of the flask when splitting or sowing out cells for experiments.

In the experimental protocols (depicted in Supplemental Figure S1) cells were seeded in 25 cm^2^-flasks in 4.5 mL culture medium containing 11 mm glucose and cultured for 3 days. Media were then renewed, and culture was continued for overnight (18 or 24 h), two (46 or 48 h) or three days (72 h) at a glucose concentration of either 11 or 27 mm. Beta-hydroxybutyrate (BHB, 5 or 20 mm) was added to this protocol in separate experiments. We used (±)-3-Hydroxybutanoic acid sodium salt from Sigma-Aldrich (product number H6501). The (±) denotes a racemic (1:1) mixture of R and S enantiomer.

Two parallel samples from each culture condition in each experiment, were prepared for oxygen consumption rate (OCR) measurements. The results from the duplicates were then averaged and defined as data representing one experiment. Cells were counted in a Countess automated cell counter.

Respirometry measurements were done basically as described^7^ using a two chamber Oxygraph-2k (OROBOROS, Innsbruck, Austria) instrument equipped with Clark polarographic oxygen sensors. The two chamber outfit restricted comparisons to conditions in one chamber compared to that of the other chamber. The parameters of oxygen consumption recorded are ROUTINE (cellular respiration supported by exogenous substrates in the culture media), LEAK (a respiration state obtained upon inhibition of ATP synthase. Reflects uncoupled respiration caused by proton leak and/or proton slip through the inner mitochondrial membrane), ETS (electronic transfer system) capacity (the maximum stimulated non-coupled respiration state obtained upon full collapse of the proton gradient across the inner mitochondrial membrane) and ROX (residual oxygen consumption that remains after inhibition of the ETS by adding rotenone and antimycin A to inhibit complex I and III respectively). Basal respiration at the ROUTINE state was recorded once a stable O_2_ flux had been obtained. Oligomycin (2 µM) was added to block the proton channel part of ATP-synthase thereby inhibiting ATP synthesis. Uncoupled respiration (caused by proton leak or/and proton slip) in the LEAK state could then be assessed. The protonophore FCCP (carbonyl cyanide-4-(trifluoromethoxy) phenylhydrazone) was titrated in steps of 1 µL or 2 µL (steps of 0.5 µM or 1.0 µM) to achieve maximum O_2_ flux, reflecting the maximum capacity of the electron transfer system, the ETS state. Finally, rotenone (0.5 µM) and then antimycin A (2.5 µM) were added to the chambers to assess residual O_2_ flux (ROX) due to non-respiratory side-reactions. ROX was corrected for by subtracting the O_2_ flux measured in the ROX state from the O_2_ flux recorded in each of the other mentioned respiratory states.

Western blotting was performed as described,^7^ by basically the same procedures as for islets (detailed below). For each experiment, two aliquots (á 5 × 10^5^ cells) from each culture condition were collected for protein extraction and further analysis in Western blotting. The results from the duplicates were then averaged and defined as data representing one experiment.

### Rat pancreatic islets

Male Sprague-Dawley rats were obtained from Scanbur (Sollentuna, Sweden). They had free access to water and a standard diet. At the time of experiments, the rats weighed 350–450 g. Islets of Langerhans were isolated by digestion with collagenase (Sigma-Aldrich) followed by sedimentation. Islets were cultured free floating, at 37°C at a humidified atmosphere of 5% CO_2_ in air in RPMI 1640 medium (Gibco, Sweden) supplemented with fetal calf serum (10%), sodium pyruvate (1 mm), L-glutamine (2 mm), penicillin (100 IU/ml) and streptomycin (100 µg/ml). The glucose concentration in the medium was either 11- or 27-mM with or without the co-presence of BHB (5 mm). Time of culture was overnight (22 h) or two days (46 h). The design is illustrated in Supplemental Fig S1.

OCR was measured in islet-capture plates of the XF96 extracellular flux analyzer (Agilent, Seahorse Bioscience, Santa Clara, CA, USA) basically as described by Joseph and coworkers.^8^ Briefly, groups of 10 size-matched islets were handpicked into individual wells of islet capture plates and incubated for 60 min at 37 °C without CO_2_ in KRH-bicarbonate free buffer containing glucose (2 mm). Additional glucose (16.7 mm), oligomycin (5 µM), FCCP (1 µM) and a mixture of rotenone and antimycin A (both 5 µM) were injected sequentially. ECAR (extracellular acidification rate) was measured in parallel by the same instrument, basically as described.^8^

Western blotting was performed as described.^9^ Briefly, islets were washed twice in ice-cold phosphate-buffered saline (PBS) and denaturized in loading buffer at room temperature for 20 min. Samples were analyzed on 12% SDS-PAGE gels run for 1 hour at 150 V before being transferred to nitrocellulose membranes for 1 hour at 250 mA. Membranes were blocked for 2 hours at room temperature with fat-free milk (5% w/v), Tween 20 (0.1%) in Tris-buffered saline, pH 7.6, and then incubated overnight at 4°C with primary antibodies for oxidative phosphorylation complexes MS604, (Mitosciences, USA). This is a “cocktail” of antibodies probing subunits for each of the complexes 1–5, these being for complex 1 NADH dehydrogenase beta subcomplex subunit 8 (NDUFB8), for complex 2 succinate dehydrogenase subunit B (SDHB), for complex 3 cytochrome b-c1 complex subunit 2 (UQCRC2), for complex 4 cytochrome c oxidase subunit 1 (MTCO1) and for complex 5 ATP synthase subunit alpha (ATP5A). Results from complex 5 were excluded since we could not establish reliable conditions to quantitate this complex. Anti-beta-actin was used as loading control. Secondary antibody incubations employed a HRP linked anti-mouse antibody for one hour at room temperature. Immunoreactive bands were visualized using chemiluminescence (ECL Western blotting reagent, Pierce, Biotechnology, USA).

ATP measurements. An ATP Bioluminescence Assay Kit (Abcam) was used. ATP was measured according to manufacturer’s instructions. Samples from each experimental condition was measured in triplicates; the mean of triplicates was entered as one observation.

ROS in culture medium and in islets was measured by an electron paramagnetic resonance (EPR) spectrometer (Bruker eScan EPR, Noxygen, Germany). CMH (1-hydroxy-3-methoxycarbonyl-2,2,5,5-tetramethylpyrrolidine) has been used to detect intracellular O⋅^−2^ in cultured cells and tissue samples; it was used as the spin probe in our experiments. Samples were incubated with CMH for 30 minutes at 37°C, frozen in liquid nitrogen, and placed into a liquid nitrogen-filled finger Dewar, which was then inserted into the Bruker eScan EPR spectrometer. The EPR spectrometer was optimized to the following parameter settings: field sweep 100 G, microwave frequency 9.76 GHz, microwave power 1.02 mW, modulation amplitude 8.3 G, conversion time 10.24 ms, number of x-scans 10 times, 512 points resolution, and receiver gain 1 × 10^3^.

## Statistics

All data are presented as mean ± SEM. A p-value <0.05 was uniformly regarded as significant.

In INS-1 832/13 cells: after checks for normality, Student’s t-test was used for comparison of paired data from culture at 27 vs. 11 mm glucose (Table 1), and Wilcoxon signed rank test was used to compare paired data from culture at 27 mm glucose and 5 mm BHB vs 27 mm glucose alone (Table 1). A Kruskal–Wallis test was used for multiple comparisons. Significance values were adjusted by Bonferroni correction (Table 2).

**Table 1.** Oxygen consumption in INS-1 832/13 cells after exposure to 27 vs. 11 mm glucose and to 27 mm glucose and 5 mm BHB vs. 27 mm glucose alone.

Oxygen consumption rate (pmol/(s*10^6^))			
	Control condition	Experimental condition	
	11 mm glucose	27 mm glucose	
Time in culture (h)	ROUTINE		*P* value
24	27.7 ± 2.4	36.8 ± 1.8	0.006
72	25.7 ± 1.0	26.6 ± 2.1	0.595
	LEAK		
24	16.5 ± 1.1	23.4 ± 1.0	0.004
72	19.3 ± 0.4	19.4 ± 1.3	0.981
	ETS		
24	62.1 ± 4.0	78.6 ± 2.9	1.000
72	65.2 ± 3.3	69.5 ± 3.7	0.288
	ROX		
24	1.9 ± 0.3	2.4 ± 0.3	0.020
72	2.4 ± 0.1	3.6 ± 0.1	0.002
	Control condition	Experimental condition	
Time in culture (h)	27 mm glucose	27 mm glucose + 5 mm BHB	*P* value
	ROUTINE		
18	37.8 ± 1.4	40.6 ± 3.0	0.219
48	45.6 ± 3.0	41.4 ± 0.2	0.563
	LEAK		
18	20.2 ± 0.6	20.9 ± 1.3	1.000
48	21.9 ± 1.5	20.7 ± 0.9	1.000
	ETS		
18	70.7 ± 1.6	74.9 ± 5.4	1.000
48	85.1 ± 4.4	74.2 ± 3.9	0.313
	ROX		
18	2.7 ± 0.2	2.8 ± 0.1	0.844
48	3.2 ± 0.1	3.3 ± 0.2	0.563

Data are mean ± SEM. The number of experiments with 27 vs. 11 mm glucose was six (24 h data) and seven (72 h data). Student’s t-test was used to calculate p-values for comparison of data (27 vs. 11 mm glucose). The number of separate experiments with 27 mm glucose and 5 mm BHB vs. 27 mm glucose alone was six (18 h data and 48 h data). Wilcoxon signed rank test was used to calculate p-values for comparison of data (27 mm glucose and 5 mm BHB vs. 27 mm glucose alone).

**Table 2.** Exposure to 27 mm glucose affects mitochondrial protein levels in INS-1 832/13 cells and in rat islets.

	Complex (C) subunit protein levels after culture at 27 mm glucose (as % of levels at 11 mm glucose)			
	INS-1 832/13 cells			
Time in culture (h)	C1	C2	C3	C4
18	80.8 ± 6.1	99.2 ± 15.2	80.5 ± 7.5	86.2 ± 9.9
*P* value	0.263	1.000	0.102	0.969
48	69.7 ± 6.3*	83.9 ± 9.1	82.2 ± 4.9	72.6 ± 9
*P* value	0.038	0.593	0.081	0.123
	Rat islets			
	C1	C2	C3	C4
22	109 ± 7.5	115 ± 8.3	119 ± 29.7	109 ± 7.1
*P* value	0.266	0.108	0.554	0.254
46	87.2 ± 5.0	92.6 ± 6.6	56.0 ± 5.2	90.0 ± 3.9
*P* value	0.033	0.242	<0.0001	0.038

INS-1 832/13: Data are mean ± SEM of six or seven separate experiments. Kruskal–Wallis test was used to calculate p-values for multiple comparisons (11 and 27 mm glucose, with and without BHB added). *p* values given in the table represent comparison of data after culture at 27mM vs 11mM glucose. Data after culture with BHB are not shown. Original Western blots are shown in Supplemental Figure S2. Rat islets: Data are mean ± SEM of eight separate experiments. None of these experiments included addition of BHB. Student’s t-test (unpaired) was used to calculate p-values for comparison of data after culture at 27 mm vs 11 mm glucose. Original Western blots are shown in Supplemental Fig. S3.

**Figure 1.**
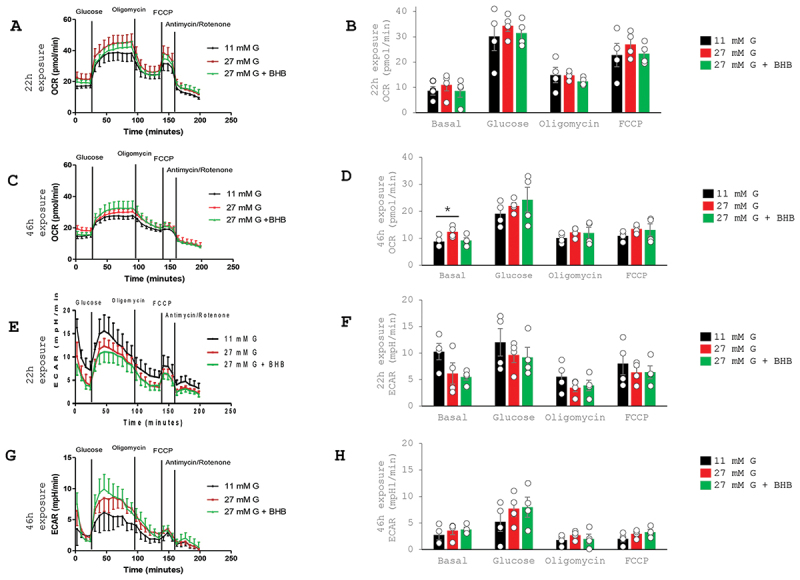
Effects of glucose on oxygen consumption rate (OCR) and extracellular acidification rate (ECAR) in rat islets. The figure shows results from measurements performed after overnight (22 h) (A, B, E and F) or two days (46 h) (C, D, G and H) of culture at 11- or 27- mM glucose. Islets of four rats were – separately for each rat – placed in parallel wells and processed in the same experiment. Measurements over time of OCR are depicted in A-B and of ECAR in E-F after overnight culture in 11- and 27-mM glucose, and for OCR in C-D and for ECAR in G-H after culture for two days. Integrated responses for basal (3.3 mm glucose) and glucose-stimulated (16.7 mm glucose) OCR are shown in C and for ECAR in D following culture overnight in 11- and 27-mM glucose, and in I and J after culture for two days. FCCP-stimulated (maximum) values of OCR are shown in E and K. Residual total OCR after oligomycin is calculated as lowest recorded value after oligomycin in % of FCCP-stimulated OCR (highest recorded value) and is shown in F (for overnight culture) and L (for two days culture). All data are mean ± SEM, n = 4. *p < .05 for differences as indicated in the figure.

**Figure 2.**
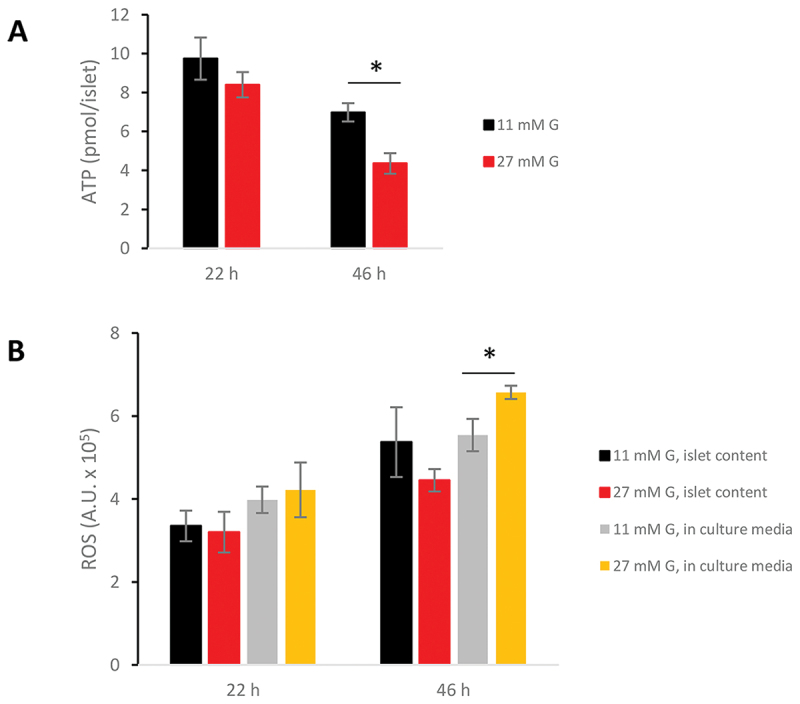
High glucose culture decreases intra-islet ATP and increases ROS in islet culture media. Electron paramagnetic resonance (EPR) was used for the detection of ROS. Results are expressed as arbitrary units (A.U.). Data on ATP and ROS are expressed as mean ± SEM of eight and four separate experiments respectively. *p < 0.05 for differences vs. levels at 11 mm glucose.

In islets: Student’s t-test was used for comparison of unpaired data (Table 2) and one-way ANOVA was used for multiple comparisons (Figures 1 and 2).

## Results

### INS-1 832/13 cells

#### OCR and Western immunoblotting

Oxygen consumption was increased after overnight culture (24 h) in 27 vs. 11 mm glucose as was the LEAK parameter (Table 1). The increase in LEAK indicates increased uncoupling. These effects by high glucose were nullified by prolonging culture to three days (72 h) (Table 1). ROX was then increased, indicating extramitochondrial participation of OCR after prolonged culture at high glucose. Addition to culture media (27 mm glucose) of BHB (5 mm) did not affect oxygen consumption (Table 1), nor did addition of 20 mm BHB, results not shown, Viability (assessed by trypsin blue and cell counting) was unaffected under all culture conditions, including exposures to BHB (results not shown).

Western immunoblotting (Table 2) demonstrated a significant decrease (after Bonferoni correction) in complex 1 after 48 of culture with high glucose. Addition to culture media of BHB did not affect the mentioned changes in complexes (p = 1.000 for effect of 48 h of exposure to 5 mm BHB together with 27 mm glucose vs. 27 mm glucose alone, data not shown). Original Western blots are shown in Supplemental Fig. S2. Results from complex 5 were excluded since we could not establish reliable conditions to quantitate this complex.

## Rat pancreatic islets

### OCR

Basal OCR and a glucose-induced response in the Seahorse protocol was unaffected after overnight (22 h) exposure to 27 vs.11 mm glucose as was also the maximal level of FCCP-induced OCR (Figure 1A,B). There were no apparent differences in the inhibitory effects of oligomycin.

Prolonging culture to two days (46 h) reduced OCR both after 11- and 27-mM exposures whereas OCR was increased during basal conditions after 27 vs 11 mm glucose (Figure 1C,D).

Addition of 5 mm beta hydroxybutyrate did not affect these patterns of response (Figure 1A-D).

### ECAR

The basal level of ECAR was unaffected after overnight (22 h) culture at 27 vs. 11 mm glucose (Figure 1E,F). Also, acute elevation of glucose in the Seahorse protocol stimulated ECAR similarly after culture at 27 vs. 11 mm glucose. Prolongation of culture led to an increase in basal ECAR and an increase in glucose induced ECAR following culture at 27 vs. 11 mm glucose (Figure 1G-H). Addition of beta hydroxybutyrate did not affect these patterns of response (Figure 1E-H).

### Western blot, ATP and ROS

Western blot data (Table 2) after overnight (22 h) culture showed non-significant changes due to high glucose. Prolonging culture to two days (46 h) led to downregulation of complex 1 and 3 and 4. Addition of beta-hydroxybutyrate did not change these findings (results not shown). Original Western blots are shown in Supplemental Fig. S3. Islet ATP levels were significantly decreased after 46 h of high glucose (Figure 2A). At the same time point were intra-islet ROS not significantly affected but moderately increased in culture media from islets exposed to 27 vs. 11 mm glucose (Figure 2B).

## Discussion

This study demonstrates in INS-1 832/13 cells a clear time dependency of alterations of OCR and complex 1 by exposure to high glucose. Results on OCR in rat islets were less striking but we found time-dependent changes on complex 1, as well as on other complexes, and also on islet ATP contents. Also, effects on ECAR measured in islets were clearly time dependent.

In pancreatic islets a modest oxidation of BHB has been reported by the use of radioactive tracers.^10,11^ Here, we probed BHB metabolism by measuring oxygen consumption directly in beta cells and islets. Exposure to 5 mm BHB together with 27 mm glucose did not significantly affect OCR whether in INS-1 832/13 cells or in islets. The absence of a clear effect by BHB may depend on the co-culture with elevated glucose. An interaction with glucose levels has been reported;^12^ further, a previous report using tracer technology found no effect by BHB in the presence of elevated glucose.^13^

The lack of significant interactions between elevated glucose and beta BHB on OCR in our study could be of clinical interest in view of BHB being the major ketone body in the circulation^5^ and present here in concentrations to be seen during diabetic ketoacidosis (taking into account that racemic BHB will yield approximately 50% of the circulating enantiomer and that the R-enantiomer is the physiologically active one).^14^ Additional data on insulin release and results from human islets would be needed before reaching a definite conclusion on clinically meaningful inhibitory interactions between glucose and ketones in beta cells.

The concept of “glucotoxicity” i.e. negative effects on beta cells by chronic hyperglycemia is well established. Multiple negative effects have been demonstrated both in vitro and in vivo. Such negative effects include dysfunctional signal -secretion mechanisms, overburdening of the endoplasmic reticulum system, mitochondrial dysfunction, the accumulation of ROS and effects on insulin biosynthesis. It seems likely that many negative effects converge to produce “glucotoxixity;” whether lipids can add to this, producing “glucolipotoxicity,” has been debated.^15^

In contrast to studies which recorded effects of long-term exposure to hyperglycemia or elevated glucose levels in vitro we sought to characterize the early time course that builds up to toxicity. Our hypothesis was that the impressive flexibility of mitochondria to changing physiological conditions would be insufficient to avoid dysfunctional effects after only a small window of functional adaptation. Our results seem compatible with this notion even if our experimental design, which takes into account the logistics of culture and time to perform measurements, does not allow us to define a precise inflection point, i.e. when adaptation changes to dysfunction.

How to explain the different effects on OCR in INS-1 832/13 cells vs. islets? One should consider that clonal cells more easily adapt to changes in metabolic environment (by inherently being more “active” and having ongoing replication) than is the case for native cells. Increased metabolism coupled with increased uncoupling may in the short – but not longer – term have prevented INS-1 832/13 cells from mitochondrial dysfunction due to over nutrition.

In rat islets we observed a decrease in OCR with time of culture per se, i.e., a decrease which occurred also after culture at 11 mm glucose. Such effects of culture have been encountered also in previous studies.^16^ A time-dependent decrease in function by culture per se could possibly be due to lack of islet vascularization leading to insufficient oxygenation.

A common feature of effects on complexes was a decrease in complex 1. A decrease in complex 1 may be in line with proteins of complex 1 being downregulated by more than 50% in the study of Haythorne et al^4^. Also, it is of interest that complex 1 is specifically degraded under conditions that result in a reduced Coenzyme Q/CoQH2 redox couple.^17^ A key role for complex 1 in regulating mitochondrial oxygen consumption seems likely in view of it being the largest of complexes and, arguably, positioned to maximally influence electron flow. In this context we note that complex 1 in islets and INS-1 832/13 cells was upregulated in a previous study, during nutrient scarcity (achieved by reduction of glucose in culture media), something which may be interpreted as an adaptive response,^18,19^

The results on ECAR in rat islets demonstrate stimulation by acutely added glucose according to the Seahorse protocol. This was surprising since the activity of lactate dehydrogenase is reportedly low in beta cells.^19^ However, interpretations of underlying mechanisms behind the dynamics of ECAR should be guarded, as emphasized by others.^20^ Hence, a proportionality of ECAR with glycolytic flux could be confounded by other sources of extracellular acidification. In any case there was a time dependency in high glucose cultured islets for a glucose-induced ECAR response suggesting a glucose-induced shift in islet metabolism. Studies are needed to establish the origin of glucose-induced perturbations of ECAR.

The measurements of ROS by CMH, a cell permeable hydroxylamine spin probe is likely to reflect overall ROS production.^21^ We note that an increase in effluxed ROS from islets evolved time dependently, similarly to mitochondrial parameters, whereas islet contents of ROS did not. We speculate that the antioxidant capacities of islets are sufficient to counteract an increased continuous load of intracellularly produced oxygen radicals, a capacity that may have increased with time of exposure to elevated glucose. Indeed, a robust ROS eliminating system has been identified in beta cells.^22^ However, verification of such a notion and possible couplings with the alterations in mitochondrial function that we observe needs further study.

## Conclusion

A high glucose environment in vitro time-dependently changes fundamental mitochondrial parameters in INS-1 832/13 cells and in rat pancreatic islets. The changes include a decrease of complex 1. Any influence of beta hydroxybutyrate on the impact of high glucose was not detected.

## Supplementary Material

Supplemental Material

Final_Suppl_Fig_3.docx

Final_Suppl_Fig_2.docx
