# Correction

**DOI:** 10.1080/19382014.2025.2557668

**Published:** 2025-09-07

**Article title:** Time dynamics of elevated glucose and beta-hydroxybutyrate on beta cell mitochondrial metabolism

**Authors:** Ik Hals, Z. Ma, M. Kylling, A. Bjørkvik, A. Zhao, S-B. Catrina, X. Zhang A. Björklund & V. Grill

**Journal:** Islets

**Bibliometrics:** Volume 17, Number 01, pages 1−9

**DOI:** http://dx.doi.org/10.1080/19382014.2025.2503515

The incorrect graphical abstract was originally published online.

The correct graphical abstract is now available in the online publication.

