# Supplementary figures and images for: Time dynamics of elevated glucose and beta-hydroxybutyrate on beta cell mitochondrial metabolism
Source: Islets. 2025 May 19;17(1):2503515. doi: 10.1080/19382014.2025.2503515 (PMC12091920; doi:10.1080/19382014.2025.2503515)

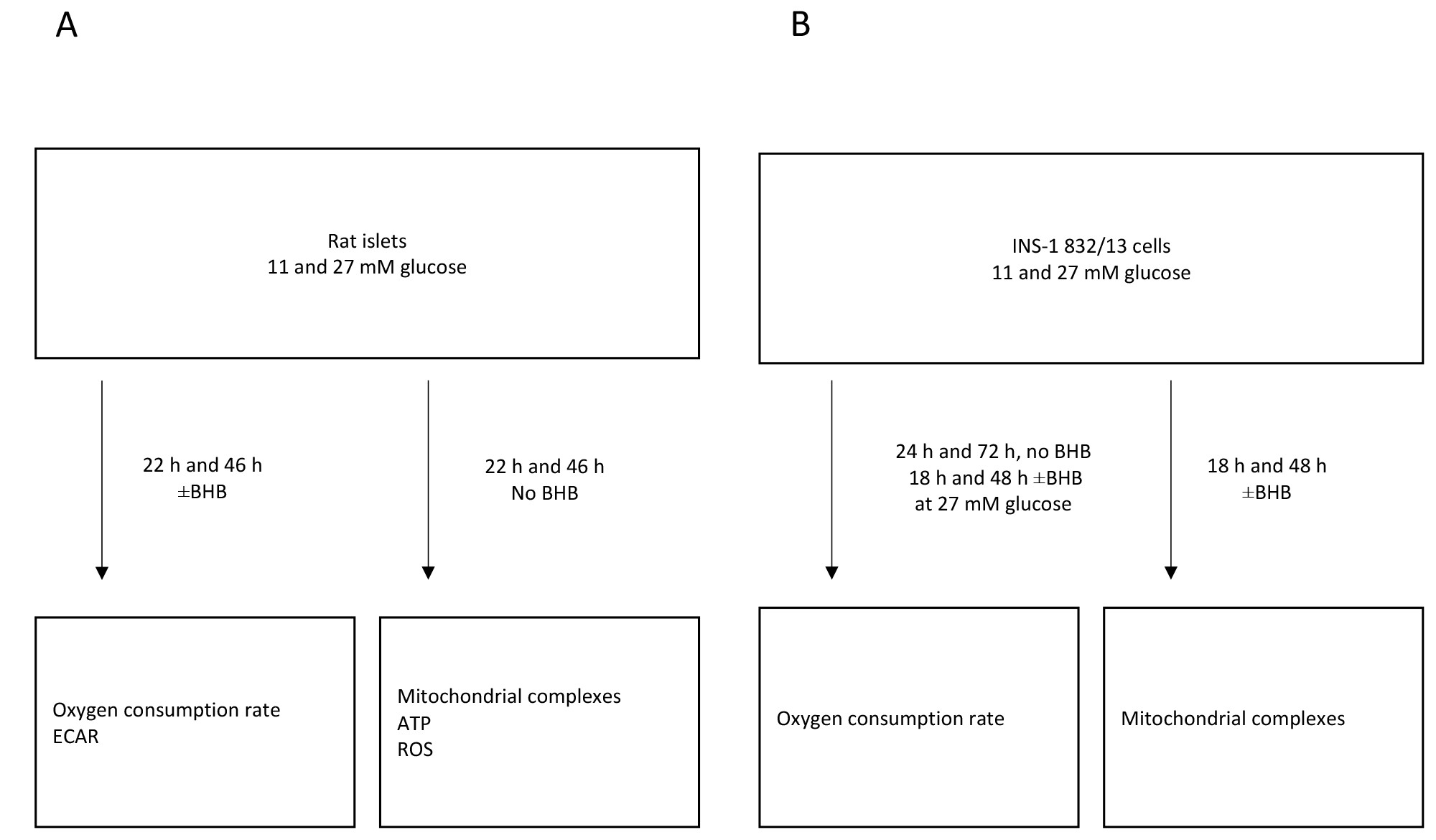

Supplement: Supplemental Material [file KISL_A_2503515_SM1995.docx]

Rat islets cultured for 22h


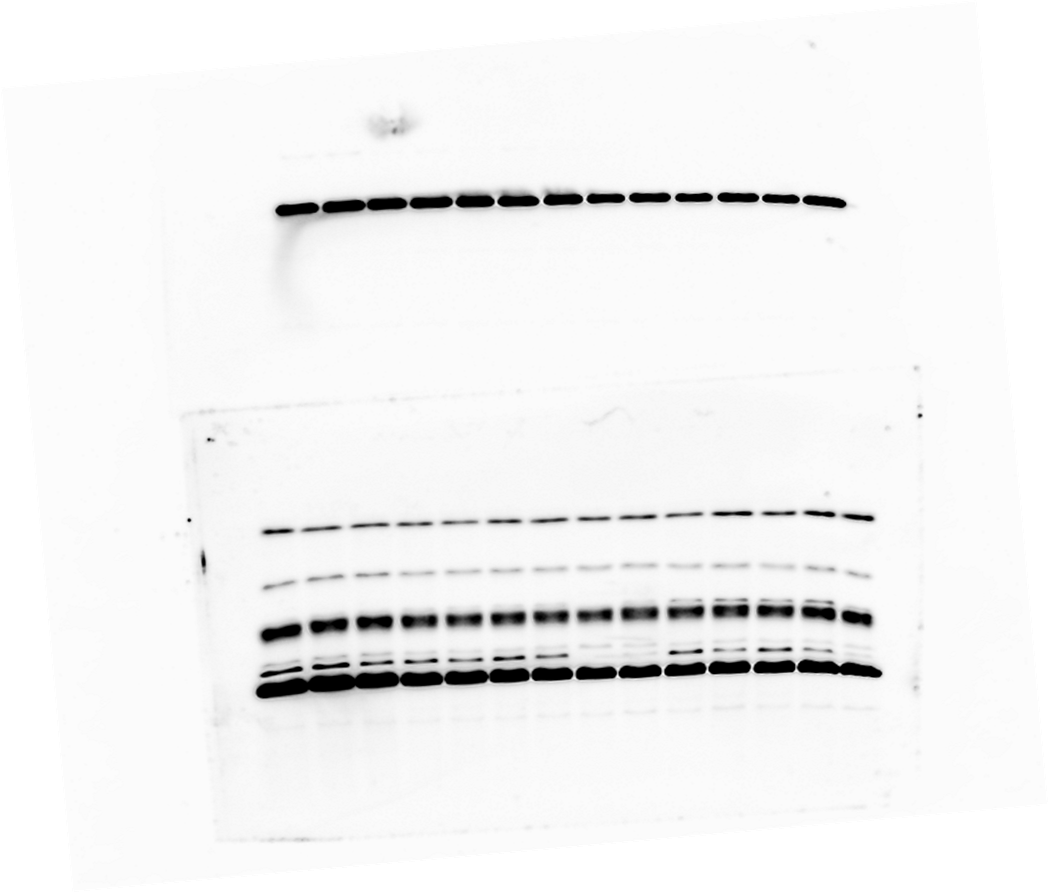
Experiment no: PC 1 2 3


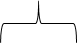

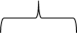


Beta-actin

CI

CII

CIV

CIII

CV

| glucose( mM) | PC | 11 | 27 | 11 | 27 | 11 | 27 |
| --- | --- | --- | --- | --- | --- | --- | --- |
| Culture (h) |  | 22 | | | | | |

Supplement: Final_Suppl_Fig_3.docx [file KISL_A_2503515_SM8131.docx]
