# Supplementary material for: Time dynamics of elevated glucose and beta-hydroxybutyrate on beta cell mitochondrial metabolism
Source: Islets. 2025 May 19;17(1):2503515. doi: 10.1080/19382014.2025.2503515 (PMC12091920; doi:10.1080/19382014.2025.2503515)
Supplement: Final_Suppl_Fig_2.docx [file KISL_A_2503515_SM8129.docx]

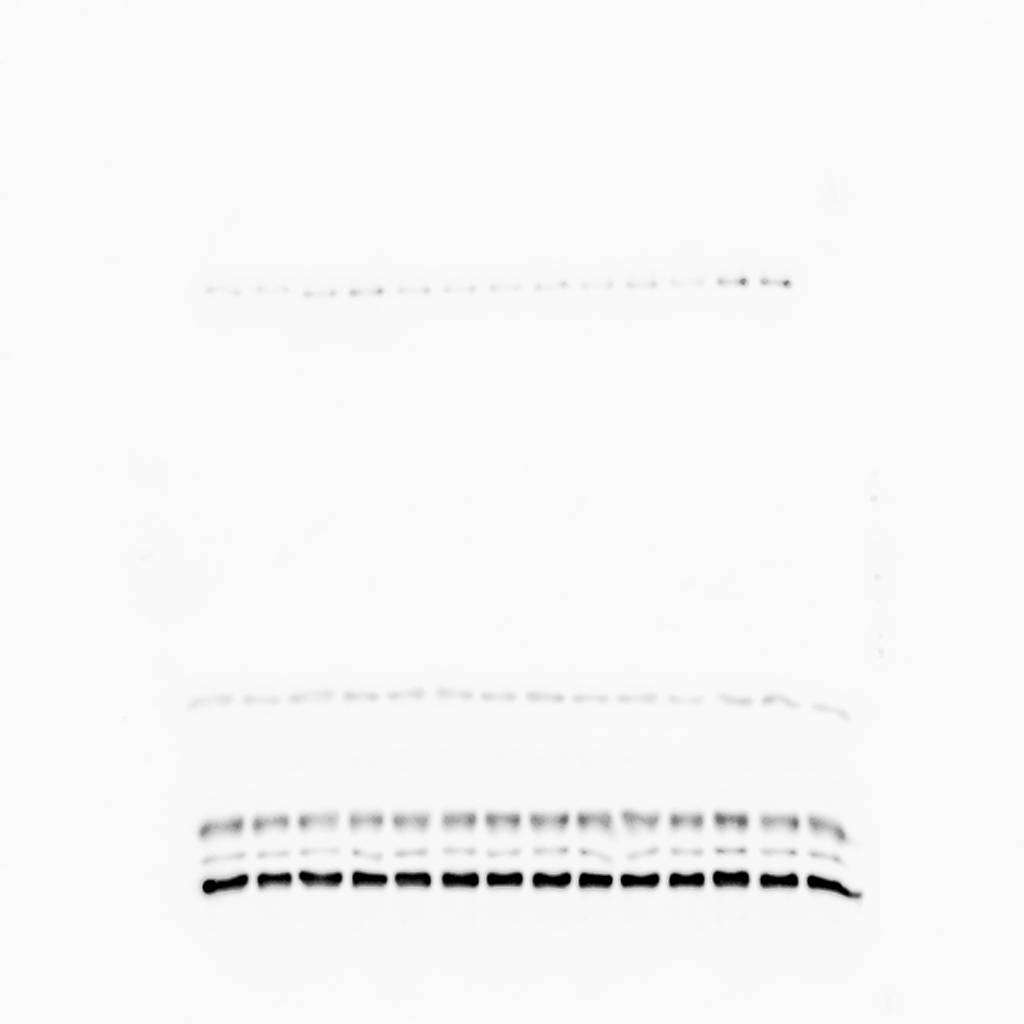


**A**

CIII

CV

Glucose (mM) PC 11 11 27 27 11 11 27 27 11 11 27 27 PC

BHB (5 mM) + + + + + +

Glucose (mM) PC 11 11 27 27 11 11 27 27 11 11 27 27

BHB (5 mM) + + + + + +

CIV

Beta-actin

CII

CI

INS-1 cell culture 18h


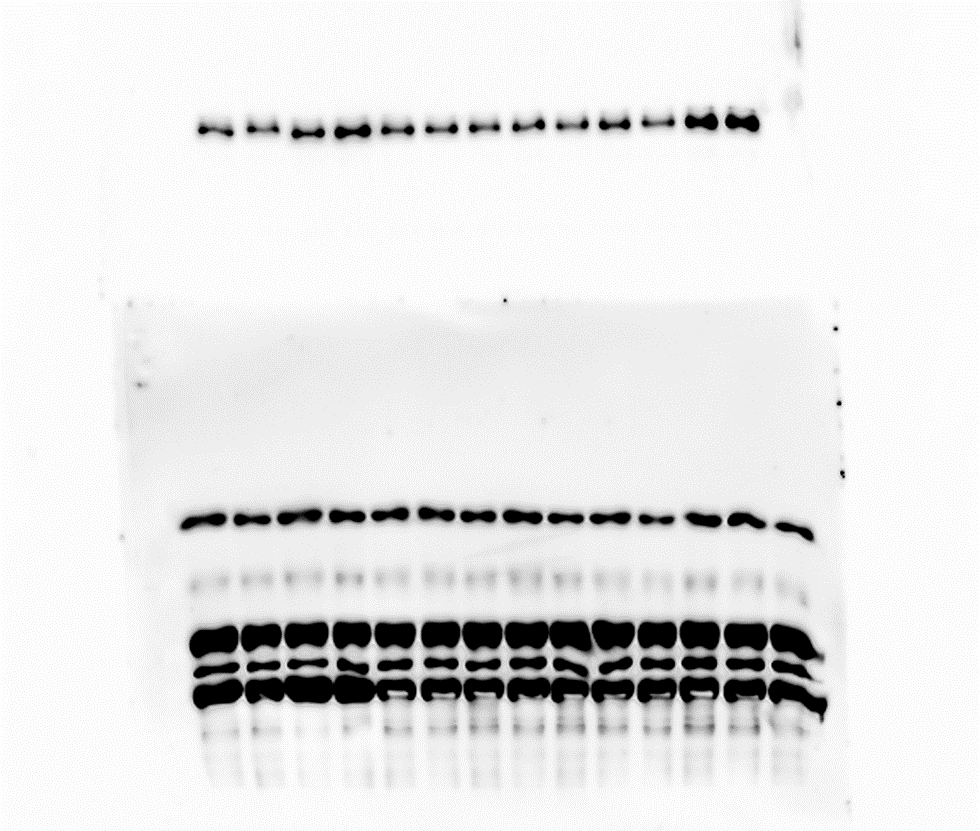


**B**

Glucose (mM) PC 11 11 27 27 11 11 27 27 11 11 27 27 PC

BHB (5 mM) + + + + + +

CII

Glucose (mM) PC 11 11 27 27 11 11 27 27 11 11 27 27

BHB (5 mM) + + + + + +

Beta-actin

CI

CIV

CIII

CV
